# Epi-regulate my brain: unlocking mechanisms of brain growth evolution

**DOI:** 10.1038/s44318-024-00083-8

**Published:** 2024-03-25

**Authors:** Virginia Fernández, Víctor Borrell

**Affiliations:** Instituto de Neurociencias, Consejo Superior de Investigaciones Científicas & Universidad Miguel Hernández, Sant Joan d’Alacant, 03550 Spain

**Keywords:** Development, Evolution & Ecology, Neuroscience

## Abstract

A new study sheds light on the mechanisms of cerebral cortex expansion during evolution.

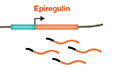

## Teaser

Humans and other great apes stand out among mammals for the big size and advanced folding of their brains’ cerebral cortex. This cortex expansion during evolution has been linked to the acquisition of higher cognitive abilities and results from gene regulatory changes during fetal development. New research in The EMBO Journal implicates the growth factor EPIREGULIN and the epigenetic regulation of its expression in the evolutionary expansion of the cerebral cortex.

Inter-species differences in cerebral cortex size primarily result from variations in the number of cortical neurons and glia. Remarkably, the basic process of cortical neurogenesis is largely conserved across mammals, and the observed variation between species reflects regulatory differences. Cortical neurons are produced by neural stem and progenitor cells (NPCs) during fetal development. These cells come in two main flavors: apical progenitor cells (APs), which make up the ventricular zone (VZ) in the inner side of the cortex, and basal progenitor cells (BPs) that form the subventricular zone (SVZ), directly above the VZ. Most APs are apical radial glia (aRG), i.e., highly proliferative neural stem cells that divide producing more aRGs, or BPs and neurons. Subsequently, BPs divide further and generate additional neurons. The proliferative and neurogenic activity of APs and BPs varies significantly across species, such that cortical expansion and folding are linked to the abundance of BP generation and expansion of their subsequent lineage. For example, in species with a smooth cortex (i.e., lissencephalic species), such as mice, APs typically generate few BPs, and these divide mostly once, generating two neurons each. In contrast, in species with a large and folded cortex (i.e., gyrencephalic species) like humans, APs produce vast amounts of BPs, which then undergo multiple rounds of amplificative cell divisions further increasing their number, to finally produce massive amounts of neurons. The increased proliferative activity of BPs in gyrencephalic organisms leads to the expansion of the SVZ and its distinction in two proliferative niches: inner and outer zones (ISVZ/OSVZ). Finally, BPs also come in different flavors, including intermediate progenitor cells (IPCs) and basal radial glia (bRG; also named outer radial glia). The relative abundance of BP types differs significantly between species, with bRGs being more prevalent in gyrencephalic species (see Del-Valle-Anton and Borrell, 2022) for a recent review).

**Figure 1:**
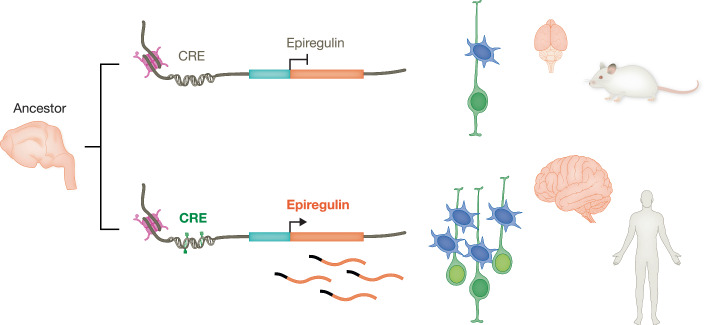
Evolution of the *EREG* locus led to brain expansion. Changes in *cis*-regulatory elements (CRE) of the EPIREGULIN gene have probably occurred after the common ancestor of mice and humans, leading to its active transcription in primate cortical progenitor cells, which massively increased proliferation resulting in the expansion of primate brain size.

Understanding the mechanisms that led to cortical expansion during mammalian evolution has gained renewed interest in the last decade, with a focus on the evolution of developmental mechanisms, particularly NPC proliferation and cell lineage. Biological evolution is based on changes and mutations in DNA and is driven by two fundamentally different mechanisms: the emergence of novel genes or gene functions (neofunctionalization), and changes in the regulation of expression of conserved genes (gene expression). The evolution of the cerebral cortex is known to have involved both mechanisms (Espinós et al, 2022). In this study, (Cubillos et al, 2024) employ cutting-edge methodology to elegantly demonstrate how both the emergence of a new gene and its transcriptional regulation contributed to the extraordinary expansion of the cerebral cortex in primates (Fig. 1). The authors delve into inter-species differences in gene expression and transcriptional regulation focused on EPIREGULIN (*EREG*), a member of the epidermal growth factor (EGF) family. EPIREGULIN is known for its involvement in various cellular processes and its multifaceted roles in cell signaling pathways. However, its role in neural progenitor cell proliferation and cortical evolution had not been explored.

Cubillos et al show that *EREG* is highly expressed in the developing fetal cortex of humans, and to a lower extent in our primate relatives, but not in the more distantly related mouse, pointing to its potential involvement in the evolution of cortical development and brain expansion (Cubillos et al, 2024). In support of this notion, the addition of exogenous EPIREGULIN to the developing mouse cortex selectively increases BP proliferation, without any effect on APs. Remarkably, this effect of EPIREGULIN is species-specific, as it also promotes BP proliferation in gorilla cerebral organoids but not in human organoids. The authors propose that either the system is saturated in the human system (BP proliferation or EPIREGULIN signaling), or human cells have a different receptivity to this growth factor. A key lesson learnt is the importance of understanding species differences in the regulation of neurogenesis, and their implications in brain development. Why is *EREG* expressed in the developing cortex of great apes but not of mice? Through the analysis of *cis*-regulatory elements and epigenetic marks in the genome, Cubillos et al identify a specific set of active *cis*-regulatory elements (CREs) associated to the *EREG* locus, which are highly conserved in primates but not in mice (Fig. 1). Intriguingly, although the sequence of these enhancers is poorly conserved, they have similar gene expression-enhancing activity in mice and humans. The identification of putative enhancer regions in the human genome associated with *EREG* expression opens up a new window of research into the epigenetic regulation of neurogenesis and its relevance to the evolution of brain complexity.

New genes provide new substrates for evolutionary innovation. Gene duplication events followed by sequence divergence lead to the birth of new genes, encoding new proteins with novel potential functions. In recent years, several studies have shown the complex genetics of the evolution of human brain development, including the identification of human-specific genes (e.g., *NOTCH2NL*), and even gene variants specific to modern humans (e.g., *TKTL1*), whose significance reverberates within the complex framework of neural evolution. Discovering the emergence of *NOTCH2NL* and its relevance to the evolutionary expansion of the human brain in two seminal studies (Suzuki et al, 2018; Fiddes et al, 2018) was a seminal milestone in our understanding of human brain evolution. The gene *NOTCH2NL* is a paralog of *NOTCH2* that distinguishes us from our closest primate relatives. Its expression exerts a profound effect on progenitor cells and cortical development, orchestrating signaling pathways that are key for neuronal differentiation. Even more strikingly, a groundbreaking study by Pinson and colleagues identified a variant of the gene *TKTL1* that is specific to modern humans, distinct from Neanderthals and any other relative (Pinson et al, 2022). The modern human variant acquired increased capability of enhancing cortical progenitor amplification, underscoring increased cortex size, which exemplifies the key relevance of modern human genetics in neurodevelopment.

Changes in the regulatory sequences of conserved genes may also profoundly shape evolutionary trajectories and influence their outcomes. Regulation of gene expression is a very strong driver of genetic diversification and phenotypic adaptation, re-using the available genetic toolkit in new ways: expression in new cell types, tissues and/or developmental epochs. Recent studies underscore the significance of differential gene regulation in shaping cortical progenitor cells and modes of neurogenesis across phylogeny (Benito-Kwiecinski et al, 2021; Cárdenas et al, 2018). Accordingly, modifications in regulatory sequences lead to divergence in gene regulatory networks, driving phenotypic diversification. Studies by Boyd (Boyd et al, 2015) and Chinnappa (Chinnappa et al, 2022) have shown how significant modifications of gene regulatory mechanisms contribute to developmental and morphological diversity.

The sophisticated research published by Cubillos et al in *EMBO J* identifies a new mechanism involved in the evolutionary divergence of neurogenesis regulation among mammals. By comparing the expression and function of EPIREGULIN in the developing cerebral cortex of humans, gorillas and mice, the study sheds new light on how differences in gene expression contribute to their diversity of neurodevelopmental outcomes. Capturing these evolutionary differences both expands our understanding of brain evolution and provides a framework for investigating the molecular mechanisms underlying species-specific brain development. These discoveries also raise new and exciting questions that warrant further investigation, including the existence of species-specific regulatory networks that fine-tune gene expression patterns during brain development. Pinpointing the intricacies of these regulatory networks, and how they evolved through phylogeny, remains of prime interest for future research. In particular, identifying the interactions of EPIREGULIN with other growth factors, and its downstream signaling pathways, will enhance our knowledge of the molecular mechanisms governing neural progenitor proliferation, and of the evolutionary expansion of the brain.
